# Mixed strategies of griffon vultures’ (*Gyps fulvus*) response to food deprivation lead to a hump-shaped movement pattern

**DOI:** 10.1186/2051-3933-1-5

**Published:** 2013-07-05

**Authors:** Orr Spiegel, Roi Harel, Wayne M Getz, Ran Nathan

**Affiliations:** Movement Ecology Laboratory, Department of Ecology, Evolution and Behavior, Alexander Silberman Institute of Life Sciences, The Hebrew University of Jerusalem, Edmond J. Safra Campus, Jerusalem, 91904 Israel; Department of Environmental Science, Policy and Management, University of California at Berkeley, Berkeley, CA 94720-3114 USA; School of Mathematical Sciences, University of KwaZulu-Natal, Durban, South Africa

**Keywords:** Fasting period, GPS-ACC tracking, Internal motivation, Movement ecology, Non-linear response, Optimal foraging, Starvation risks, Supplementary feeding management, Vulture conservation

## Abstract

**Background:**

The need to obtain food is a critical proximate driver of an organism’s movement that shapes the foraging and survival of individual animals. Consequently, the relationship between hunger and foraging has received considerable attention, leading to the common conception that hunger primarily enhances a “food-intake maximization” (FIMax) strategy and intensive search. A complementary explanation, however, suggests a trade-off with precautions taken to reduce the risk of physiological collapse from starvation, under a strategy we denote as “energy-expenditure minimization” (EEMin). The FImax-EEmin trade-off may interact with the forager’s hunger level to shape a complex (non-monotonic) response pattern to increasing hunger. Yet, this important trade-off has rarely been investigated, particularly in free-ranging wild animals.

We explored how hunger affects the movements of adult griffon vultures (*Gyps fulvus*) in southern Israel. Transmitters combining GPS and accelerometers provided high-resolution data on vultures’ movements and behavior, enabling the identification of feeding events and the estimation of food deprivation periods (FDPs, measured in days), which is used as a proxy for hunger.

**Results:**

Data from 47 vultures, tracked for 339 ± 36 days, reveal high variability in FDPs. While flight speed, flight straightness and the proportion of active flights were invariant in relation to food deprivation, a clear hump-shaped response was found for daily flight distances, maximal displacements and flight elevation. These movement characteristics increased during the first five days of the FDP sequence and decreased during the following five days. These characteristics also differed between short FDPs of up to four days, and the first four days of longer FDP sequences.

These results suggest a switch from FIMax to EEMin strategies along the FDP sequence. They also indicate that vultures’ response to hunger affected the eventual duration of the FDP. During winter (the vultures’ incubation period characterized by unfavorable soaring meteorological conditions), the vultures’ FIMax response was less intensive and resulted in longer starvation periods, while, in summer, more intensive FIMax response to hunger resulted in shorter FDPs.

**Conclusions:**

Our results show a flexible, non-monotonic response of free-ranging wild animals to increasing hunger levels, reflecting a trade-off between increasing motivation to find food and the risk of starvation. The proposed trade-off offers a unifying perspective to apparently contradictory or case-specific empirical findings.

**Electronic supplementary material:**

The online version of this article (doi:10.1186/2051-3933-1-5) contains supplementary material, which is available to authorized users.

## Background

The movement of individual organisms is a key component of many ecological and evolutionary processes across a broad range of spatiotemporal scales, reflecting a dynamic interplay of factors that are both internal and external to the focal organism [1]. Internal factors include hunger, thirst, fear and sex drive, while external factors include habitat structure and locations of key resources (e.g., water, refuge) and of predators and enemies [1, 2]. Identifying the role of intrinsic motivations is particularly challenging due to its hidden and stochastic nature, and observed movement patterns may reflect the combined effects of several non-mutually-explicit motivations [3, 4]. For instance, the switch that ladybird beetles make from an extensive to an intensive search mode after encountering a food item, could result from intrinsic motivation (satiation level) or from external factors related to resource patch distribution [5]. Whereas it is often impossible to determine internal motivation directly, it can be inferred indirectly, for example, by comparing individuals in different contexts or different situations for the same individuals [2, 6–8].

The need to obtain food or other essential resources is a major internal driver of various ranging movements referred to as foraging. For an animal with a given set of internal states (e.g., physiological or reproductive status) and constraints (motion and navigation capacities), foraging movements are generally subject to a basic trade-off between food intake rate and locomotion costs such as energetic expenditure, time allocation and predation risk. The degree of satiation (or hunger) an animal experiences at a given time can profoundly impact the internal state and the constraints, as well as the outcomes of these trade-offs [9–11]. For instance, hungry animals may be more prone than satiated animals to take risks [9, 12, 13], and they may alter their movement patterns and prey selection criteria [2, 6–8].

Although the effect of hunger on diet breadth has received considerable attention in the optimal foraging literature [14, 15], there appears to be a lack of theoretical assessment of the expected effect of hunger on movement or, more generally, on activity. It is commonly accepted that high hunger levels of active foragers searching for unknown food items generally leads to more intensive search, aimed at “*food-intake maximization*” (FIMax). This strategy may be reflected in either longer search time and distance [8, 11, 14] or in a switch to an area-restricted search where resources are clumped [7, 8]. Despite the general finding of such a monotonic response, we suggest that hungry animals facing the risk of starvation and physiological collapse may switch to an alternative strategy aimed at “*energy-expenditure minimization*” (EEMin). This strategy can lead to a non-monotonic effect of starvation on movement such as the unimodal responses found in some experimental studies [6, 10].

Notably, in contrast to other aspects of optimal foraging and response to starvation, the vast majority of studies examining the interplay between starvation and movement have been conducted in confined experimental arenas and have focused on relatively small-bodied species, including insects [7, 8, 16], spiders [11, 17], snails [18] and fishes [10, 13, 19]. Although experimental designs ensure controlled assessment of the hunger level, they might not well represent natural scenarios. Since hunger level can affect foraging movements in various ways, depending on the study system, species, and body size, this relationship should also be explored in large-bodied species and for free-ranging individuals under natural conditions [11]. Addressing this challenge requires quantification of hunger level and movement patterns of free-ranging animals. This task has become feasible recently through newly developed research tools, including high-resolution accelerometers and GPS data loggers, combined with machine learning algorithms and remote sensing tools [20].

Vultures are large obligate scavengers that rely on thermal and orographic uplift for their soaring flight that enables them to cover long distances in search of carcasses [21]. Because carcasses are an irregular and unpredictable resource, vultures frequently experience long intervals between successive feedings and are adapted to cope with fasting periods of up to two weeks without detrimental effects [22, 23]. Field studies have shown that griffon vultures (*Gyps fulvus*) regularly fast for several days between successive meals. Ten-day laboratory starvation experiments have also demonstrated that they are capable of saving energy by decreasing their metabolic rate and modifying thermoregulation [22, 24]. The high mobility, fasting ability and easily quantified food resources of vultures make them an attractive species to study the effects of hunger on movement. Human activities have caused a major decline in vulture species worldwide, intensifying the use of supplementary feeding stations (SFSs) as a common management practice. This practice can alter the feeding and foraging patterns of vultures [25–27], providing further motivation for studies of the interactions between food satiation and movement in vultures.

We hypothesize that vultures may respond to increasing hunger by alternating their foraging strategy, leading to non-monotonic changes in their movement pattern along a starvation event. Among many possible responses reflecting various combinations of differentially weighted FIMax and EEMin effects, a hump-shaped movement pattern is expected if vultures switch from FIMax to EEMin strategy in response to increasing starvation. The FIMax strategy should lead hungry individuals to forage more intensively, fly for longer durations and cover longer distances than satiated individuals. Yet, the EEMin strategy, expected to be more pronounced as the starvation period becomes very long, should lead these *very* hungry vultures to reduce activity levels while waiting for socially-transferred information on food availability at a nearby SFS or at an occasional carcass elsewhere [25, 27]. We used biotelemetry tags to quantify the movements and behaviors of free-ranging griffon vultures in order to test this hypothesis and the expected hump-shaped movement pattern. The tags enabled us to identify feeding events and determine the time elapsed since the last feeding event, used as a proxy for hunger level, together with detailed information on the vultures’ movement pattern.

## Methods

### The study system and tracking protocols

The Eurasian griffon vulture (*Gyps fulvus*, Hablizl 1783) is an obligatory scavenger that relies on carcasses as food sources [28, 29]. Griffons form colonies on cliffs where they roost and breed. The breeding cycle starts with egg-laying in early winter (December–January), continues with rearing the chicks through spring and early summer; ends when chicks fledge around August [30]. In Israel and its surroundings, the species was once a common resident throughout the mountainous regions. However, during last decades it has been declining steadily; recent surveys by the Israeli Nature Protection Authority (NPA) estimate the current population size to be roughly 250 individuals. Today, most individuals persist in small colonies in gorges in the Judean and Negev deserts in southern Israel, as well as in south-western Jordan, with some immigration to and from the northern parts and other countries [31, 32].

**Figure 1:**
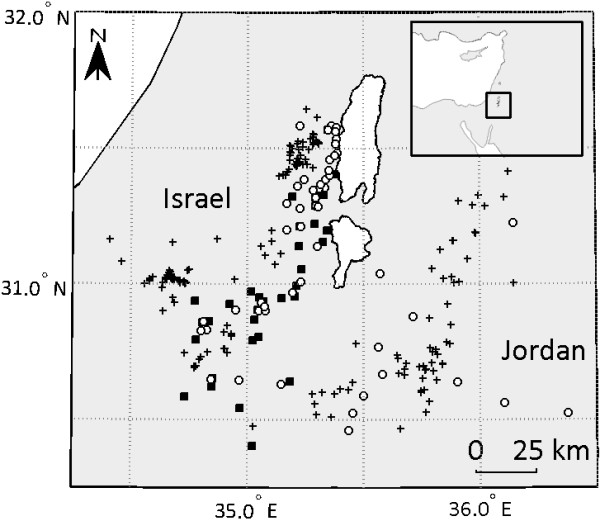
**A map of the study area, showing the location of vultures’ roost sites (open circles), supplementary feeding stations (SFSs; black squares) and feeding events at occasional carcasses (black crosses).** Each FDP sequence starts with a feeding event and ends one day before the successive feeding event.

Changes in land use during the past six decades have limited the number of naturally-occurring carcasses in southern Israel to the point that the area no longer sustains a viable population of vultures. Therefore, the NPA routinely performs supplementary feeding in southern Israel as a major vulture management practice. Ungulate carcasses (cows, calves, camels and goats) are constantly provided at approximately 25 SFSs across the region, accounting for 25.4 ± 1.0 feeding events per month (Figure 1). These feedings supply 9246 ± 380 kg month^-1^, satisfying the population’s food requirements. However, since food is distributed stochastically among SFSs (with some SFSs used more frequently than others, as in the French “light feeding stations” [27]), and since carcass existence time is short (either due to rapid decomposition or consumptions by scavengers and carnivores), uncertainty regarding food availability forces vultures to actively search for food. Indeed, vultures are also frequently observed feeding on occasional carcasses outside the SFSs (see also Results), indicating they do not solely rely on the SFSs but actively search for rather unpredictable food resources as well. Monitoring and trapping using walk-in traps, another component of NPA vulture management, results in approximately 120 birds year^-1^ being captured during the non-breeding season (September to November) and marked with metal and colored rings and patagial tags.

During the vulture trapping campaigns 2008–2011, we equipped 53 adult (>4 years old) vultures with GPS/ACC telemetry tags (E-Obs GmbH; Munich, Germany) that had three independent functional components: (1) a GPS device that provided the three dimensional position and the instantaneous ground speed; (2) an accelerometer that measured acceleration (ACC) at three perpendicular axes at 3.3 Hz for 20 s each; and (3) a pinger that emitted a tag-specific UHF-signal. The pinger helps in detecting a tagged bird from up to 30 km under ideal conditions, facilitating the downloading of stored GPS and ACC data through UHF communication (typically at distances of approximately 1–3 km). The tags were fitted to the vultures in a backpack configuration and weighed ~190 g (2.4–3.5% of the vultures’ body mass, harness included). In general, tags were set to sample GPS locations and ACC measurement at 10 min intervals during a 12 h diurnal duty cycle and missing points were interpolated, accounting for ~10% of the non-static points. There were minor variations in the sampling protocol among tags and years see [20] for details.

The research was conducted with permission from the NPA (2008/31144) and the ethics committee of the Hebrew University (NS-07-11063-2).

### Data processing, feeding events identification and food deprivation periods (FDPs)

Daily movement characteristics were analyzed from the GPS data. On ~10% of the days, the vultures did not travel more than 2 km from their initial location (the roost) and these days were considered “inactive”. For “active” days, the “roost departure time” was calculated as the time interval from sunrise to the first non-static point (instantaneous ground speed >4 m s^–1^). On ~9% of the days, the vultures left earlier than the first GPS sample (07:00 or 06:30 h). The “daily travelled distance” was calculated by summing all distances between successive points in the same day, and the “maximal displacement” was defined as the Euclidean distance between the initial and farthest location of the day. Because vultures typically return to the same or nearby roost each day, we defined flight “straightness” as the ratio between travel distance and the displacement from the initial and farthest locations of each day. Travel speed refers to average flight speeds, calculated as the daily mean of GPS instantaneous ground speeds of all non-static points. The daily path was also scanned for day-stops, defined as locations at which the vulture landed and remained static within a 400 m radius for more than 20 min. Vultures usually made one or two stops per day, and rarely more than four in addition to the final stop at the evening roost.

We applied ACC-based classification of behavioral patterns for griffon vultures. This rapidly evolving method in movement ecology has been described in detail elsewhere [20] and was used in the present study to identify feeding events from vultures’ tracks. Each ACC measurement was classified into one of seven behaviors: active flight (wing flapping), passive flight (soaring–gliding), standing, lying down, preening, running (including other active behaviors on the ground), and eating. Classification was performed using an artificial neural networks-supervised machine-learning algorithm and a large training set of ground-truthed behaviors. 

For the purposes of the current study, it was essential to correctly identify unobserved feeding events during which the vulture actually fed. Consequently, the artificial neural network algorithm was chosen over slightly better performing ones (e.g., Random Forest) since it provided the most accurate classification of the “eating” behavior that is critical for our study [20]. To further minimize false positive errors, we applied a set of conservative filters to identify *feeding* events, rather than indiscriminately using all cases that the classification algorithm identified as “eating”. Therefore, we only considered eating cases in which the vulture was on the ground, but not within major roost sites where carcasses were very unlikely to occur. In addition, vultures usually spend a few hours at a carcass site (mean ± SE: 02:45 ± 00:03, *n* = 2509), sometimes up to a whole day, and only rarely (<2%) less than 40 min. Vultures at the carcass site are passive most of the time, with short bursts of eating and fighting over the carcass. Therefore, we defined a feeding event as occurring when: (i) more than one ACC measurement during the stop was classified as “eating” (93.3% of classified events); (ii) a single “eating” event was accompanied by two or more “running” cases (indicating fight and/or hop) within the same stop (1.9% of classified events); or (iii) a single “eating” case occurred in a specific time and location when and where other tagged vultures were eating (0.9% of classified events). Stops at which a single “eating” observation was accompanied by a single “running” measurement were classified as “undecided” (3.8% of the classified events).

**Figure 2:**
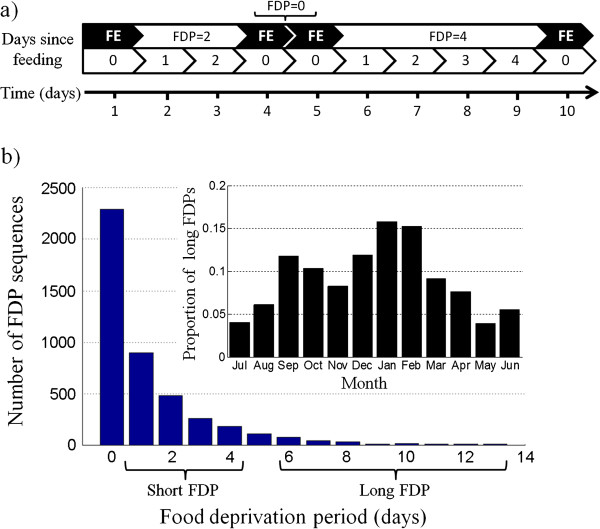
**Definition and occurrence of food deprivation periods (FDPs) in griffon vultures.** **(a)** A schematic illustration of a timeline with feeding events (FE, marked in black) and FDPs to clarify terminology used throughout the paper. Each FDP sequence starts with a feeding event and ends one day before the successive feeding event. For instance, a FDP of zero represents feeding events on consecutive days. **(b)** A histogram of FDP for 4397 feeding events identified from the movement tracks of 47 vultures tracked with GPS/ACC tags. The inset shows the proportion of monthly events with FDP ≥ 6 days.

The food-deprivation period (FDP; measured in days) preceding each feeding event is termed herein simply as “days since feeding” of that individual (feeding days themselves are not counted, as illustrated in Figure 2a). If this period contained days with missing data, or a day with a stop classified as “undecided”, this feeding event was excluded from further analysis. The few cases (1%) with FDP longer than two weeks, which might represent rare transmitter malfunctioning, were considered as unrealistic and omitted from further analysis. We explored the distribution of FDP in our dataset, as well as the seasonal distribution of short (FDP of 1–4 days) and long (FDP ≥6 d) FDP sequences.

### Data analysis

To examine whether movement patterns differ between hungry and satiated vultures, we compared several characteristics of the movement track recorded a day before and a day after a given feeding event while controlling for multiple comparisons with Benjamini- Yekutieli’s correction procedure [33] (see Additional file 1 for further details).

To examine the potential effects of FDP length on movement, we averaged all values of the *i*^th^ day along the FDPs from all the feeding events of each vulture; this avoided pseudo-replication and temporal autocorrelation among different events of the same individual. Sequences of FDP > 10 were excluded from this analysis due to insufficient sample size (Figure 2b). To evaluate the relationship between each movement characteristic (response variable *Y*) and FDP (explanatory variable *X*), we considered three alternative effects of FDP: No effect (*Y = C*), a linear effect (*Y = bX + a*), and a hump-shaped effect (*Y = aX*^*2*^ *+ bX + c*). For each characteristic, we selected the best fitting model using Akaike’s information criterion with a correction for small sample size (AIC_c_). Selection of a hump-shaped effect may imply that vultures switch from an FIMax to an EEMin strategy after a certain hunger threshold has been reached (in our case around five days of not feeding). Higher order polynomial fits (e.g., cubic) were not considered here primarily because they cannot be justified by simple *a-priori* theoretical considerations.

To further test the flexibility of vultures’ responses to FDPs, we repeated the same analysis separately for different subsets of the data. First, since SFSs are known to have pronounced effect on vultures’ movement [27], and might also affect the way vultures response to hunger, we divided the entire dataset into two subsets of events that ended either at an SFS or at an occasional carcass (Additional file 2). Second, we compared subsets of short and long FDP sequences (feeding events preceded by 1–4 or ≥6 days of no feeding) and compared the vultures’ movements between the two sequence types (i.e., between the short event and the first four days of long events). Similar values for the two would imply that vultures follow one strategy (FIMax) during early stages of FDP (i.e. 1–4 in our case) regardless its final length. Significant differences in vultures’ movement patterns between short and long sequences, however, would suggest more complex responses of vultures to hunger. In addition to alternating between FIMax and EEMin strategies *along* the FDP sequence, such differences suggest that vultures may alternate among strategies also at the *early* stages of the FDP sequence and that strategy choice during these stages eventual influences the resulting FDP duration. Therefore, we applied two-way repeated measures ANOVA with vultures as subjects and FDP sequence category (short or long) and days as factors to test for movement pattern differences. All computations were carried out in Matlab (MathWorks, Natick, Massachusetts, USA).

## Results

GPS/ACC data were retrieved from 47 of the 53 vultures, with tracks of 339 ± 36 days (ranging from 30 to 1264 days), reflecting 15,966 vulture days with 57.8 ± 3.4 locations daily and *ca.* 1.22 million data points in total. Approximately one-third of the days (5386) included one or more stops with a feeding event, suggesting that vultures in our study system eat on average roughly every three days. Most of the feeding events (71.4%) occurred at an SFS. The remaining feeding events were at occasional carcasses indicating that vultures routinely search for rather unpredictable food resources away from the SFSs (Figure 1). Feeding events typically start at 09:35 ± 00:06 in the morning and 27% of the stops with “eating” activity started before 7:00 am. We filtered out events in which the preceding FDP length could not be assured, using the remaining 4397 events with known FDP for analysis. The statistical distribution of FDP closely followed a negative exponential function (*Y* = 2257*e*^-0.*8x*^, *R*^2^ = 0.99; Figure 2b), with 52% of feeding events in consecutive days (FDP = 0) and the remaining 2113 events with FDP ≥ 1. The seasonal distribution of long FDPs peaks to 15% of the monthly events during the winter (January–February), and reaches a minimum of approximately 5% during early summer (May–June). A secondary peak was observed during the autumn (September–October) (Figure 2b; inset).

Vultures were more active when they were hungry (Additional file 1: Figure S1), that is, during the day preceding a feeding event. When hungry, vultures flew significantly longer distances and moved farther away from the roost at higher altitudes than when satiated (see Additional file 1: Table S1 for details). Hungry vultures also showed a trend of earlier departure from their roost, although the latter result was not significant after multiple comparison correction. Flight speed, flight straightness and proportion of flapping flights did not differ between hungry and satiated vultures.

**Figure 3:**
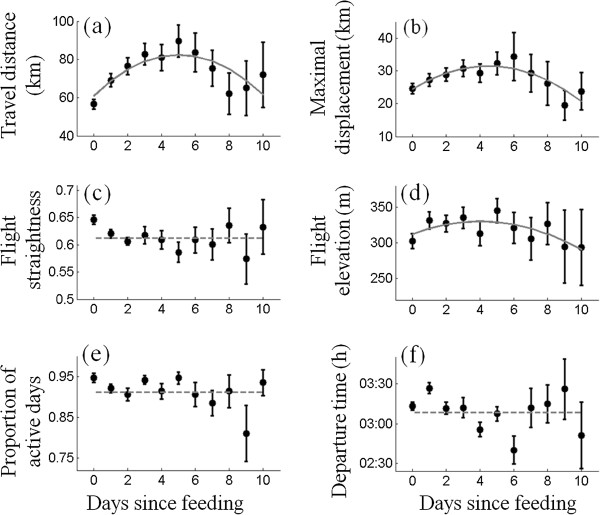
**The change in various movement characteristics of vultures as a function of time since the last feeding event (the days within a FDP).** The lines represent the best fitting model among a hump-shaped model (second-order polynomial, grey solid line), a linear response model (first-order polynomial; green dash-dotted line) and a lack of response model (grey dashed line on the mean value). Models were ranked according to AIC_c_. Travel distance **(a)**, maximal displacement **(b)** and flight elevation **(d)** showed similar hump-shaped trends, increasing for the first five days and decreasing afterward. FDP length had no clear effect on flight straightness **(c)**, proportion of active days **(e)** and roost departure time **(f)**. Error bars are S.E. between individual mean values.

Changes in vulture movement characteristics were associated with changes in FDP length (Figure 3), irrespective of the number of daily fixes or the proportion of interpolated points. A hump-shaped response was the best fitting model for the travel distance, maximal displacement and flight elevation (Figure 3a,b,d; see Additional file 3: Table S2 for details), suggesting that vultures modify their behavior along the FDP sequence. The inference about the apparent increase of travel distance on the ninth and tenth days (Figure 3a) should be made with caution due to the very limited sample size for these long events (see Figure 2b). No clear trend was observed for flight straightness (vultures’ average daily straightness was 0.617 throughout the entire range of FDP; Figure 3c), proportion of active days (vultures were active on 90% of the days, regardless of the time since last feeding; Figure 3e) and roost departure time (vultures typically depart their roost 3 h after sunrise; Figure 3f). Very similar results were obtained for a subset of 3150 events ending at an SFS (Additional file 2). The hump-shaped response was also obtained for a subset of 1247 events ending at occasional carcasses; the analysis of this subject revealed similarities in our results as well, and also some differences, but all were rather minor (Additional file 2: Figure S2).

**Figure 4:**
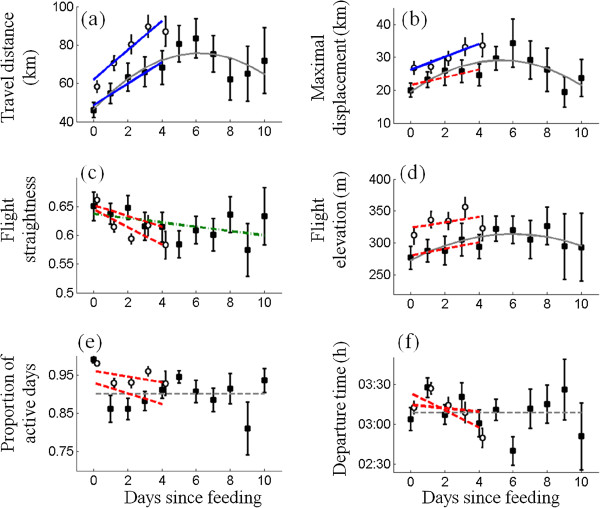
**The change in various movement characteristics of vultures as a function of time since the last feeding event (the days within a FDP) during short (1 ≤ FDP ≤ 4 d, white circles; shifted right for clarity) and long (FDP ≥ 6, black boxes) sequences.** Long grey lines represent the best fitting model for the subset of long FDP sequences (see caption of Figure 3 for further details). Short thick lines represent linear regression for the short sequences and the corresponding period of the first four days of long sequences. Solid blue and dashed red lines are regression fits with slopes significantly or not significantly different than zero, respectively. FDP has a hump-shaped effect on daily travel distance **(a)**, max displacement **(b)** and flight elevation **(d)**; a linear effect on flight straightness **(c)** and no clear effect on proportion of active days **(e)** and roost departure time **(f)**. Short sequences differ from the corresponding period of long sequences for all characteristics excluding flight straightness **(c)** and roost departure times **(f)**.

The vultures’ behavior also differed significantly between short (*n* = 1810) and long (*n* = 188) FDP sequences. For the subset of long sequences, FDP had a hump-shaped effect on daily travelled distance (Figure 4a; see Additional file 3: Table S3 for details), maximal displacement (Figure 4b) and flight elevation (Figure 4d), suggesting that vultures change their movement during long sequences. FDP had a linear but negligible effect on flight straightness (Figure 4c), and other characteristics showed no significant trends for the entire period. Overall, vultures flew 193.3 ± 9.5 km during short sequences and 599.4 ± 58.3 km during long events. Feeding events following both FDP sequence types had similar probabilities of occurring at an SFS (0.69 and 0.61, respectively) rather than at an occasional carcass.

We found a significant difference in the response of vultures to short FDP sequences and the corresponding period (i.e., the first four days) of the long FDP sequences. For the paired design of the repeated measures ANOVA, we used a subset of the vultures (*n* = 20) that had at least three long sequences and three sequences of FDP = 4 days. Significant associations with sequence type were found for daily travel distances (F_1,4_ = 26.5, *p* < 0.0001), maximal displacement (F_1,4_ = 21.9, *p* = 0.0002), flight elevation (F_1,4_ = 6.9, *p* = 0.017) and activity level (F_1,4_ = 11.9, *p* = 0.003). No difference was found for flight straightness and roost departure time. A significant effect of FDP duration (the day factor) was found for daily travel distances (F_4,1_ = 5.9, *p* < 0.0001) and activity level (F_4,1_ = 8.6, *p* < 0.0001), while a somewhat marginal effect was found for roost departure time (F_4,1_ = 2.5, *p* = 0.048). The interaction term between date and sequence type was insignificant for all characteristics, suggesting that the direction of vulture response to FDP did not differ between the groups.

## Discussion

We used high-resolution GPS/ACC tracking to quantify how the change in the internal state of an animal (here hunger level) shapes its movement patterns. As expected, we found that griffon vultures alter their movement patterns when hungry. The finding that several important movement characteristics changed in a hump-shaped pattern along the food deprivation period (FDP), and differed between short and long FDP sequences were less trivial. Vultures increased their daily flight distances during early FDP stages and even more so in short FDP sequences, while decreasing them at later stages of FDP. This implies that vultures combine different strategies in the face of increasing hunger and that these strategies affect ensuing FDP lengths.

Because for active foragers both food detection probability (i.e., expected benefits) and travel costs are positively correlated with distance, we suggest that the general hump-shaped response revealed in this study reflects a trade-off between the need to find food (necessitating longer flights) and the need to conserve energy (necessitating shorter flights). The first strategy can be viewed as “food-intake maximization” (FIMax) and the second as “energy-expenditure minimization” (EEMin). The expectation that both external (e.g., season) and internal factors (e.g., variable energetic needs during the breeding cycle) may interact in shaping flexible response to hunger is supported by the clear seasonal pattern in the prevalence of different foraging strategies (and the consequent FDP durations) observed in this study.

### Vultures’ hump-shaped response to FDP agrees with optimal foraging considerations

Our general finding that changes in hunger level are significantly associated with changes in animal behavior and movement agree with the general theoretical expectation that increasing hunger levels should alter animal behavior [14] and with studies of other animals [2, 6–8, 10, 11, 16, 17]. We found no effect of hunger on flight speed or the proportion of active flights, indicating that energy expenditure (per unit time) during flight is relatively constant and that flight distance is a good proxy for invested time and energy. This finding also implies that both aspects are rather conservative, and reflect constraints in the ability of the species to alternate its behavior under normal circumstances. In contrast, we found that when hungry, vultures are more active, depart earlier from their roost and cover longer distances than when satiated (Additional file 1: Figure S1), suggesting that vultures who search for sparsely distributed carcasses (either occasional or artificially supplied) invest more in foraging [21, 28].

Interestingly, travel distance, maximal displacement and flight elevation show a hump-shaped response to FDP – increasing during the early stages and decreasing during the advanced stages of the FDP sequence – which implies that vultures exhibit a qualitative shift in their response to hunger as FDP grows (Figure 3). The prediction that growing hunger should increase an animal’s motivation to find food [10, 14, 34] and reduce travel costs (due to reduction of body mass [35]) is supported by the observed FIMax strategy, where distances increase during the early stages of the sequence. Soaring (the primary flight mode of vultures) is considered to be energy-efficient, but the energetic costs associated with flight increase with flight distance [21]. Hence, the opposing trend during advanced stages of the FDP sequence could be explained by the trade-off with the increasing need to conserve energy and minimize starvation risks, resulting in an EEMin strategy [10, 34]. Focusing on a subsets of the data representing only long FDP sequences (Figure 4) or only events ending at occasional carcasses (Additional file 2: Figure S2) reinforces this general result: During long FDP sequences, and also when feeding on occasional carcasses, the hump-shaped response of these movement characteristics is similar to the pattern observed in the entire data set. Therefore, it is unlikely that this finding of a behavioral shift is an artifact generated by a mixture of multiple causes, or by foraging behavior that is aimed at artificially supplied food only.

This behavioral shift between the two strategies, observed after approximately five days of starvation, agrees with findings of physiological experimental studies in our focal species, revealing a sharp decrease in body mass and metabolic rate during the first five days (Phase I), followed by a much slower body mass loss and constant metabolic rate during the rest of the experiment (Phase II; days 5–10) [22]. After 10 days, vultures were presumably still far from entering a renewed phase of decreased metabolism and rapid mass loss (Phase III) that indicates detrimental effects that can reduce viability and ultimately cause death [22]. These results agree with our findings for free-ranging birds: Despite the negative trend after prolonged starvation (FDP ≥ 6 days), vultures maintained high activity levels (>85% of the days) and travelled relatively long distances each day (~50 km).

### The hump-shaped effect of hunger on movement as a general pattern unifying previous results

Despite the general finding that hunger level affects movement and that hungry animals are more active, studies differ substantially regarding the direction of this effect. Some studies found that hunger increases travel distances and movement tortuosity e.g., [7, 11], while others found the opposite trend e.g., [3, 16]. A switch to area-restricted search was suggested as evidence for higher motivation to find food in predatory bugs foraging for patchy resources [7, 8]. However, resources in our study system are stochastically distributed among sparse, conspicuous SFSs (or even less predictably for occasional carcasses that constitute ~29% of all feeding events); this suggests that longer flight distances for vultures increase the likelihood of food detection [21, 28, 36]. Observed differences among studies may indeed reflect differences among species and systems, but such differences could also arise from methodological limitations. In many studies, the effect of hunger was estimated by comparing the behavior of experimentally starved and satiated animals, usually involving two and rarely more than four different discrete treatments. Such experimental designs are unlikely to identify unimodal responses when they exist. More flexible designs that are able to treat hunger as a variable taking on a range of values (at least five or more) are needed to identify non-monotonic behavioral responses that result from trade-offs among various competing factors.

A few studies have comprehensively assessed the effects of hunger on movement by using more than two FDPs. Sogard and Olla [10] found a clear unimodal response to increasing FDP using six experimental FDPs for juvenile walleye pollock fish and suggested that the fishes alternated between foraging strategies – increasing searching behavior at intermediate levels and switching to energy conserving mode at higher FDP levels. Henaut et al. [6] compared a few indices of movements by predatory bugs starved for two, six, nine and 12 hours in an experimental arena, revealing a hump-shaped response with strongest effect at intermediate starvation levels. Lamine et al. [8] repeated a similar protocol using a few life stages of another predatory bug with five FDPs of up to 48 hours, and also found complex effects on movement indices with remarkable differences among stages.

### Differences between movement strategies during short and long FDP sequences

We found a significant difference in the movement patterns of short and (the corresponding period of) long sequences. Had we found no differences between the two types, our conclusion would have been that vultures have one typical response to FDP that includes the FIMax for the early phases and a shift to EEMin after starvation for approximately five days. The observed differences, however, suggest that vultures’ response to increasing FDP is more flexible and includes also two distinct *intensities* of the former strategy (differing in amplitude but not in the trend), applied by vultures facing different FDPs (Figure 4). Rather than suggesting that vultures can anticipate the expected duration of a coming FDP and act accordingly, we propose that the observed differences in vultures’ movements between short and long FDPs imply that vultures’ behavior affects the eventual length of the FDPs at least to some extent.

The first, more intense type of FIMax strategy (observed in short FDPs) includes high activity levels, relatively long travel distances and displacements, and a tendency to depart earlier with increasing FDP. Here vultures invest high (and increasing) effort in searching that presumably facilitates food finding and leads to shorter search periods, making this type of FIMax the prevailing strategy among short FDP sequences. In contrast, the alternative “low level” type of response, observed in the *early days* of long FDPs, is characterized by shorter flight distances and lower activity levels that increase moderately with FDP; it lacks the trend of earlier departure time (excluding day zero – the day of the feeding event, where vultures were obviously active and therefore activity levels were high). Here, despite the moderate increase in search effort during days 1–4, the vultures’ activity remains at low levels, thereby spending less energy on active search. This less intensive search, in turn, results in longer times until food is found, and therefore leads to longer FDP sequences that often include the EEMin strategy at advances stages.

Vultures’ need to conserve energy during long FDPs is emphasized by the three times longer cumulative travel distance during long events (~600 km vs. 200 km during short events). Vultures are known to rely on social information for food finding [29, 37], and the combination of the “low level” FIMax strategy followed by EEMin can be effective if they wait in the vicinity of their roost for reliable social information about available food elsewhere. Moreover, given the supplementary feeding management in our study site, a vulture may be patrolling along a limited set of nearby SFS, located within less than 30 km from its current roost [27, 37]. It may be argued that the hunger effects reported here cannot be generalized to regions without SFSs, and that the switch to EEMin strategy after a certain starvation period might not occur elsewhere. However, the hump-shape response found for the entire dataset was prominent also after restricting the analysis to vultures feeding on occasional carcasses and not in the SFSs. Nevertheless, further tests are needed to evaluate how general our findings are in other species and systems.

### FDP and seasonal variability affect the foraging strategies of the vultures

Why do vultures display these two distinct foraging strategies? Foraging strategies may be age- and sex-dependent [9]. The first aspect cannot be tested in our dataset, which includes only adult birds. The second aspect can be rejected since we found no difference between sexes in the proportion of long FDP sequences (data not shown). The seasonal variability in the proportion of long FDP sequences, with high values (low feeding intensity) during early autumn and late winter (Figure 2), may be explained by two non-mutually-exclusive effects: season-specific environmental conditions and breeding phase-specific needs and constraints. In autumn, adult vultures engage in pre-breeding movements and tend to leave their core home range [Spiegel et al., *in preparation*] and cross through regions where food might be scarcer (without SFSs), forcing them to reduce feeding intensity during this season. Few individuals also exhibit long-range forays in autumn [20], further intensifying this trend. Winter conditions in the study region are characterized by short days and unfavorable thermal conditions that may deter vultures’ ability to cross long distances [20]. During this period, breeding vultures incubate their single egg (for ~55 days), presumably leading to reduced activity and energetic needs, as reflected in the frequent occurrence of long FDP sequences in this season. In contrast, the high feeding frequency during May (i.e., the smaller proportion of long FPDs) corresponds well with the most critical period of the reproductive cycle when nestlings (typically 6–10 weeks old) grow at their fastest rate [38]. Summer months also offer favorable thermal conditions that facilitate soaring flights and hence the intense FIMax strategy.

### Methodological limitations and alternative explanations

In contrast to experimental manipulation, our methodology does not provide information on the quantity and quality of consumed food. Vultures’ hunger levels may vary substantially after a feeding event. Yet, our reliable data on the lack of feeding enable addressing the main hypothesis of this study; regardless of the additional uncertainty associated with meal size, hunger levels invariably increase as FDP lengthen. Additional data on exact hunger levels (or at least consumed food) could improve our ability to explain the remaining observed variance in the response of the vultures. Although we found no association between the length of the current and previous FDPs, hunger might also vary due to recent feeding history rather than exclusively depending on the timing of the last feeding event.

Alternative explanations for the observed differences in vulture movement characteristics between the two FDP sequence types could be partly attributed to our indirect proxy for hunger. What appears to be an intense FIMax strategy may actually reflect a hungry vulture whose current hunger level corresponds to a longer FDP than that estimated by our proxy. The difference in travel distances for day zero agrees with this alternative explanation: While distances are very short for the long sequences, they are substantially higher for short ones, possibly suggesting that vultures were hungrier already at this phase. Nevertheless, such a directional bias, whereby stops classified as feeding events are consistently a product of false-positive classification errors, is extremely unlikely considering the high accuracy of the classification algorithms, the conservative estimation of feeding events based on “eating” ACC measurements, and the consistency of the hump-shaped response for long FDPs.

## Conclusions

Most previous studies of the effect of FDP on movement were indoor experiments on a limited set of small-bodied animals. Our study presents one of the first attempts to address this challenge in free-ranging animals that move over large areas on a daily basis. Our results reveal a broad flexibility in the behavioral mechanisms used by griffon vultures to cope with increasing hunger levels. The initial response to food deprivation is increased activity and movement, indicative of greater searching efforts and attempt to maximize food intake. A switch to energy-conserving strategy is evident in extended FDPs. These results agree with previous empirical evidence, and offer a unifying perspective to apparently contradictory (or case-specific) findings. Another level of flexibility in the vultures’ response to increasing hunger level is reflected in their ability to vary the intensity of a food-intake maximization strategy. Less intensive efforts resulted in longer FDPs and were more common during the winter and autumn, reflecting seasonal breeding cycle and environmental conditions. Vultures’ flexibility might reflect an adaptive response to unpredictable resources and should be considered by managers when applying supplementary feeding programs. For instance, a feeding regime during spring should be more intensive since vultures’ feeding intensity and activity levels during this period are higher.

## Availability of supporting data

Additional file 1: Comparing hungry and satiated vultures.

Additional file 2: FDP effects on vulture movement associated with feeding on occasional carcasses.

Additional file 3: Tables S2-S3. The effect of FDP lenght on vulture movement characteristics.

## Electronic supplementary material

Additional file 1: **Comparing hungry and satiated vultures.** (DOCX 101 KB)

Additional file 2: **FDP effects on vulture movement associated with feeding on occasional carcasses.** (DOCX 136 KB)

Additional file 3: **The effect of FDP length on vulture movement characteristics (Tables S2-S3).** Three alternative models were considered: No effect, a linear effect, and a hump-shaped effect. The tables present the best fitting model for each variable using AICc and adjusted R^2^ estimation of goodness of fit. Table S2 presents the effect for the whole dataset and Table S3 for a subset of long FDPs (>=6). (DOCX 16 KB)

## Abbreviations

ACC: Accelerometer
EEMin: Energy-expenditure minimization
FDP: Food deprivation period
FIMax: Food-intake maximization
GPS: Global positioning system
NPA: Nature protection authority
SFS: supplementary feeding station

## References

[1] Nathan R, Getz WM, Revilla E, Holyoak M, Kadmon R, Saltz D, Smouse PE (2008). A movement ecology paradigm for unifying organismal movement research. Proc Natl Acad Sci USA.

[2] Wallin H, Ekbom B (1994). Influence of hunger level and prey densities on movement patterns in three species of ***Pterostichus*** beetles (Coleoptera: Carabidae). Environ Entomol.

[3] Patterson TA, Thomas L, Wilcox C, Ovaskainen O, Matthiopoulos J (2008). State-space models of individual animal movement. Trends Ecol Evol.

[4] Holyoak M, Casagrandi R, Nathan R, Revilla E, Spiegel O (2008). Trends and missing parts in the study of movement ecology. Proc Natl Acad Sci USA.

[5] Biesinger Z, Haefner JW (2005). Proximate cues for predator searching: a quantitative analysis of hunger and encounter rate in the ladybird beetle, *Coccinella septempunctata*. Anim Behav.

[6] Henaut Y, Alauzet C, Lambin M (2002). Effects of starvation on the search path characteristics of *Orius majusculus* (Reuter) (Het., Anthocoridae). J Appl Entomol.

[7] Claver MA, Ambrose DP (2003). Influence of hunger level and prey density on searching behaviour of the reduviid predator *Rhynocoris marginatus* (Fabricius) (Het., Reduviidae). J Appl Entomol.

[8] Lamine K, Lambin M, Alauzet C (2005). Effect of starvation on the searching path of the predatory bug *Deraeocoris lutescens*. Biocontrol.

[9] Stephens DW, Krebs JR (1986). Foraging Theory.

[10] Sogard SM, Olla BL (1996). Food deprivation affects vertical distribution and activity of a marine fish in a thermal gradient: potential energy-conserving mechanisms. Mar Ecol Prog Ser.

[11] Walker SE, Marshall SD, Rypstra AL, Taylor DH (1999). The effects of hunger on locomotory behaviour in two species of wolf spider (Araneae, Lycosidae). Anim Behav.

[12] Gilliam JF, Fraser DF (1856). Habitat selection under predation hazard: test of a model with foraging minnows. Ecology.

[13] Morgan MJ (1988). The influence of hunger, shoal size and predator presence on foraging in bluntnose minnows. Anim Behav.

[14] Charnov EL (1976). Optimal foraging: attack strategy of a mantid. Am Nat.

[15] Ebersole JP, Wilson JC (1980). Optimal foraging: the responses of *Peromyscus leucopus* to experimental changes in processing time and hunger. Oecologia.

[16] McIntyre NE, Wiens JA (1999). Interactions between landscape structure and animal behavior: the roles of heterogeneously distributed resources and food deprivation on movement patterns. Landsc Ecol.

[17] Persons MH (1999). Hunger effects on foraging responses to perceptual cues in immature and adult wolf spiders (Lycosidae). Anim Behav.

[18] Stenton-Dozey JME, Brown AC, O’Riain J (1995). Effects of diet and starvation on feeding in the scavenging neogastropod *Bullia digitalis* (Dillwyn). J Exp Mar Biol Ecol.

[19] Croy M, Hughes R (1991). The influence of hunger on feeding behaviour and on the acquisition of learned foraging skills by the fifteen-spined stickleback, *Spinachia spinachia* L. Anim Behav.

[20] Nathan R, Spiegel O, Fortmann-Roe S, Harel R, Wikelski M, Getz WM (2012). Using tri-axial acceleration data to identify behavioral modes of free-ranging animals: general concepts and tools illustrated for griffon vultures. J Exp Biol.

[21] Ruxton GD, Houston DC (2004). Obligate vertebrate scavengers must be large soaring fliers. J Theor Biol.

[22] Bahat O: *Physiological adaptations and foraging ecology of an obligatory carrion eater – the griffon vulture (Gyps fulvus)*. : Tel Aviv University, Department of Zoology; 1995. [*PhD thesis*]

[23] Prinzinger R, Nagel B, Bahat O, Bogel R, Karl E, Weihs D, Walzer C (2002). Energy metabolism and body temperature in the griffon vulture (*Gyps fulvus*) with comparative data on the hooded vulture (*Necrosyrtes monachus*) and the white-backed vulture (*Gyps africanus*). J Ornithol.

[24] Bahat O (1998). Long-range movements of griffon vultures from Israel. Torgos.

[25] Deygout C, Gault A, Sarrazin F, Bessa-Gomes C (2009). Modeling the impact of feeding stations on vulture scavenging service efficiency. Ecol Model.

[26] Carrete M, Lambertucci SA, Speziale K, Ceballos O, Travaini A, Delibes M, Hiraldo F, Donázar JA (2010). Winners and losers in human-made habitats: interspecific competition outcomes in two Neotropical vultures. Anim Conserv.

[27] Monsarrat S, Benhamou S, Sarrazin F, Bessa-Gomes C, Bouten W, Duriez O (2013). How predictability of feeding patches affects home range and foraging habitat selection in avian social scavengers?. PLoS One.

[28] Houston DC (1974). Food searching in griffon vultures. Afr J Ecol.

[29] Mundy P, Bunchart D, Ledger J, Piper S (1992). The Vultures of Africa.

[30] Mendelssohn H, Leshem Y, Wilbur SR, Jackson JA (1983). Observations of reproduction and growth of old world vultures. Vulture Biology and Management.

[31] Schohat E, Ovadia O, Hatzofe O (2012). Estimating Griffon Vulture (Gyps fulvus) Survival in Israel Based on Mark-Resight Data.

[32] Le Gouar P, Rigal F, Boisselier-Dubayle MC, Sarrazin F, Arthur C, Choisy JP, Hatzofe O, Henriquet S, Lécuyer P, Tessier C, Susic G, Samadi S (2007). Genetic variation in a network of natural and reintroduced populations of griffon vulture (*Gyps fulvus*) in Europe. Conserv Genet.

[33] Benjamini Y, Yekutieli D (2001). The control of the false discovery rate in multiple testing under dependency. Ann Stat.

[34] Wieser W, Krumschnabel G, Ojwang-okwor JP, Zoologie Z, Innsbruck U, Innsbruck A (1992). The energetics of starvation and growth after refeeding in juveniles of three cyprinid species. Environ Biol Fishes.

[35] Witter MS, Cuthill IC (1993). The ecological costs of avian fat storage. Philos Trans R Soc Lond B Biol Sci.

[36] Spiegel O, Getz WM, Nathan R (2013). Factors influencing foraging search efficiency: why do scarce Lappet-faced vultures outperform ubiquitous White-backed vultures?. Am Nat.

[37] Deygout C, Gault A, Duriez O, Sarrazin F, Bessa-Gomes C (2010). Impact of food predictability on social facilitation by foraging scavengers. Behav Ecol.

[38] Xirouchakis SM, Mylonas M (2006). Status and structure of the griffon vulture (*Gyps fulvus*) population in Crete. Eur J Wildl Res.

