# Supplementary material for: Mixed strategies of griffon vultures’ (Gyps fulvus) response to food deprivation lead to a hump-shaped movement pattern
Source: Mov Ecol. 2013 Jul 5;1(1):5. doi: 10.1186/2051-3933-1-5 (PMC4337378; doi:10.1186/2051-3933-1-5)
Supplement: Supplementary file 1 — Additional file 1: Comparing hungry and satiated vultures. (DOCX 101 KB) [file 40462_2013_5_MOESM1_ESM.docx]

**Additional file 1: Comparing hungry and satiated vultures**

To examine whether movement patterns differ between hungry and satiated vultures, we compared several characteristics of the movement track recorded one day before a given feeding event and on the day after the event. Prior to the paired design *t*-test, all events of each individual vulture were averaged to a single value, thereby avoiding pseudo-replication problems. A minimal FDP (food deprivation period) threshold of two days was applied in order to focus on cases for which vultures were more likely to be hungry on the day preceding a feeding event, and to avoid the possibility that the day after the *n*-th feeding event would be the same as the day preceding the *n*+1 event. We also checked the consistency of the results for three- and four-day minimal FDP thresholds. To control for false discovery rate in testing eight characters we applied Benjamini-Yekutieli’s correction procedure setting of *P* = 0.0184 as the H_0_ rejection criteria (Benjamini & Yekutieli, 2001).

Vultures were more active when they were hungry, that is, during the day preceding a feeding event (figure S1a; see Table S1 for details) and they also showed a trend of earlier departure from their roost (figure S1b), although the latter result was not significant after multiple comparison correction. Despite the similar flight speed (figure S1c), flight straightness (figure S1d) and proportion of flapping flights (figure S1e), hungry vultures flew longer distances (figure S1f) and moved farther away from the roost (figure S1f) at higher altitudes (figure S1h) than satiated vultures. Repeating the analysis for minimal thresholds of FDP of three or four days (1217 and 736 feeding events, respectively) produced similar results: Hungry vultures had a significantly longer travel distance and daily displacement, although the trend of higher activity levels and flight elevation becomes insignificant with these higher FDP thresholds.


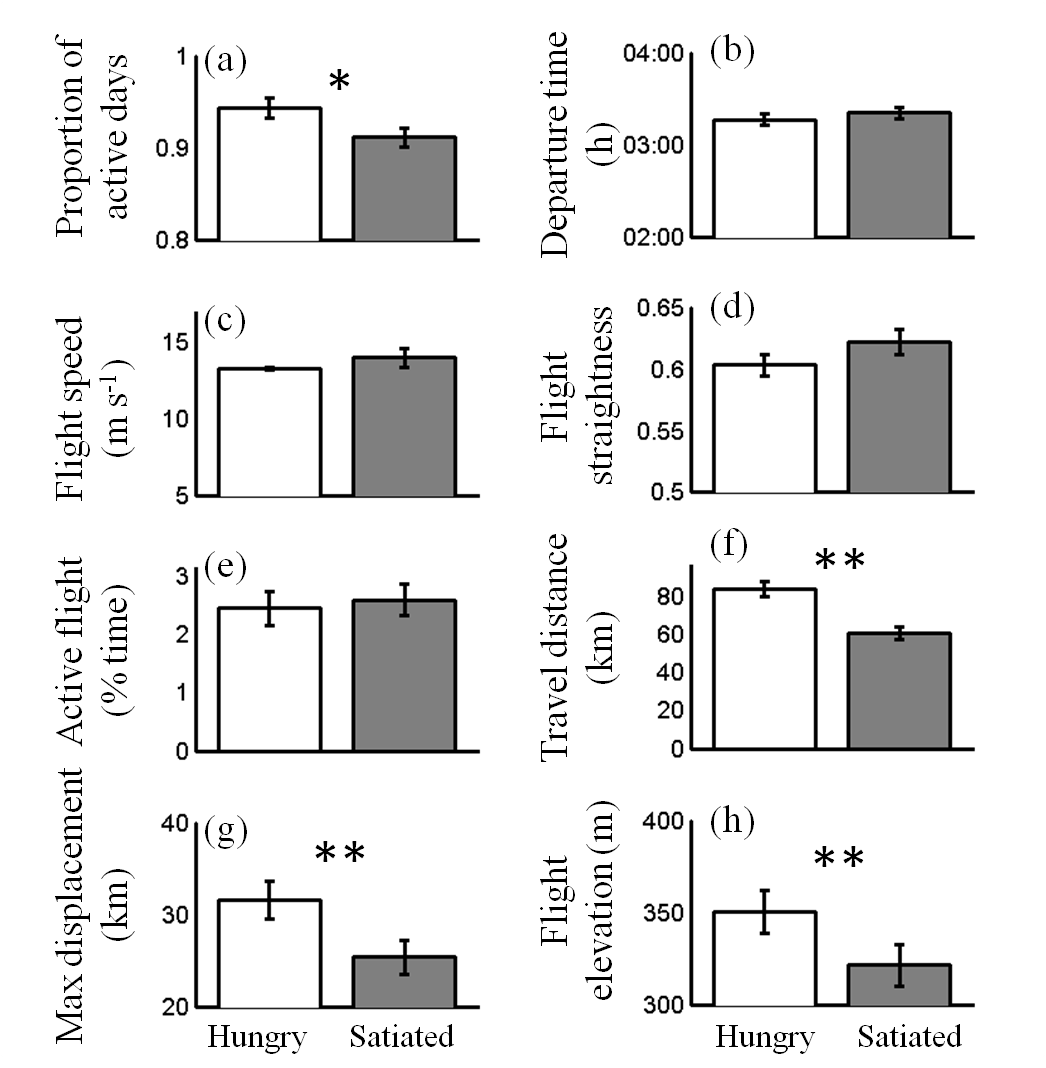


**Figure S1**. Comparison of daily movement patterns between hungry and satiated vultures. The bars represent averages for the day before a feeding event (white, hungry) and the day after that event (grey, satiated). Only events that were preceded by at least two days of food deprivation were included (*n*=1967). One and two asterisks represent significant differences after the Benjamini-Yekutieli correction with *p*< 0.018 and *p*< 0.001, respectively. Error bars are S.E. between individual mean values.

**Table S1**. Comparison of movement characteristics between hungry vultures (one day before a feeding event after at least two days without eating) and satiated vultures (one day after the same feeding event). Differences in **bold** are significant after a multiple comparison correction (see text for details).

| Variable (*x*) | Day before | Day after | *T* _46_= | *p*-value |
| --- | --- | --- | --- | --- |
| Activity level (% of active days) | 94 ± 1.1 | 89 ± 1.5 | 2.6 | 0.011 |
| Roost departure time (h after sunrise) | 03:09 ± 00:03 | 03:23 ± 00:04 | 2.3 | 0.023 |
| Flight speed (m s^-1^) | 13.2 ± 0.1 | 14.1± 0.9 | 1.1 | 0.231 |
| Flight straightness | 0.599 ± 0.01 | 0.624 ± 0.01 | 1.6 | 0.108 |
| Active wing flapping (%) | 2.5 ± 0.3. | 2.6 ± 0.3 | 0.5 | 0.582 |
| Daily travel distance (km) | **89.8 ± 4.1** | **60.3 ± 3.6** | **9.6** | **<0.0001** |
| Maximal displacement (km) | **33.8 ± 2.0** | **25.9 ± 2.1** | **5.1** | **<0.0001** |
| Flight elevation (m above ground) | **350 ± 12** | **323 ± 11** | **2.8** | **0.007** |

**References**:

Benjamini, Y. and Yekutieli, D. 2001. **The control of the false discovery rate in multiple testing under dependency**. *Annals of Statistics*, **29**(4): 1165–1188.
