# Supplementary material for: Mixed strategies of griffon vultures’ (Gyps fulvus) response to food deprivation lead to a hump-shaped movement pattern
Source: Mov Ecol. 2013 Jul 5;1(1):5. doi: 10.1186/2051-3933-1-5 (PMC4337378; doi:10.1186/2051-3933-1-5)
Supplement: Supplementary file 2 — Additional file 2: FDP effects on vulture movement associated with feeding on occasional carcasses. (DOCX 136 KB) [file 40462_2013_5_MOESM2_ESM.docx]

**Additional file 2: FDP effects on vulture movement associated with feeding on occasional carcasses**

Roughly 70% of the feeding events in our dataset are from vultures eating at supplementary feeding stations. Since feeding stations are known to modify vulture foraging movements (Monsarrat et al. 2013) there was a need to test whether our findings of hunger effects on vulture movements could be generalized to regions without supplementary feeding stations. We therefore repeated the same analysis of the entire dataset reported in the main text for two subsets, a subset of 3150 events of vultures eating in a feeding station, and a subset of 1247 events of vultures feeding from occasional carcasses outside the feeding stations.

The results for the feeding stations subset (not shown), were strictly similar to the result of the entire data set reported in the main text: travel distance, maximal displacement and flight elevation showed a hump-shaped response and no clear effect of FDP on for the three other characteristics (activity level, flight straightness, and roost departure time).

The results for the occasional carcasses subset (figure S2) were rather similar to the general results (figure 3), but some differences were also found. Agreement with the entire dataset was found for the hump-shaped response for maximal displacement (figure S2c vs. figure 3c), for flight elevation (figure S2d vs. figure 3d), and for the lack of effect of FDP on roost departure time (figure S2f vs. figure 3f). The differences were the U-shaped pattern for flight straightness (Figure S2c) and the weak negative linear effect on the proportion of active days (figure S2e); both showed no effect in the entire dataset (figures 3c and 3e, respectively). Another difference found was the lack of effect on the travel distance (figure S2a), compared with the hump-shaped effect in the entire dataset (figure 3a). We note that these disagreements might be attributed to uncertainties associated with the small number of events of rare long FDPs. For instance, the relative high daily travel distance on the 10^th^ day was due to two birds (one event each) with mean values of >200km, while for all other birds (n=8) travel distance was <120 km for the 10^th^ day; analysis of the same subset after removing only these two outliers yielded a hump-shaped pattern (second-order polynomial gave the best fit), similar to the pattern found for the entire dataset.

**References**:

Monsarrat, S., Benhamou, S., Sarrazin, F., Bessa-Gomes, C., Bouten, W. and Duriez, O. 2013. **How predictability of feeding patches affects home range and foraging habitat selection in avian social scavengers?** *PloS one*, **8**: e53077–53085.

**
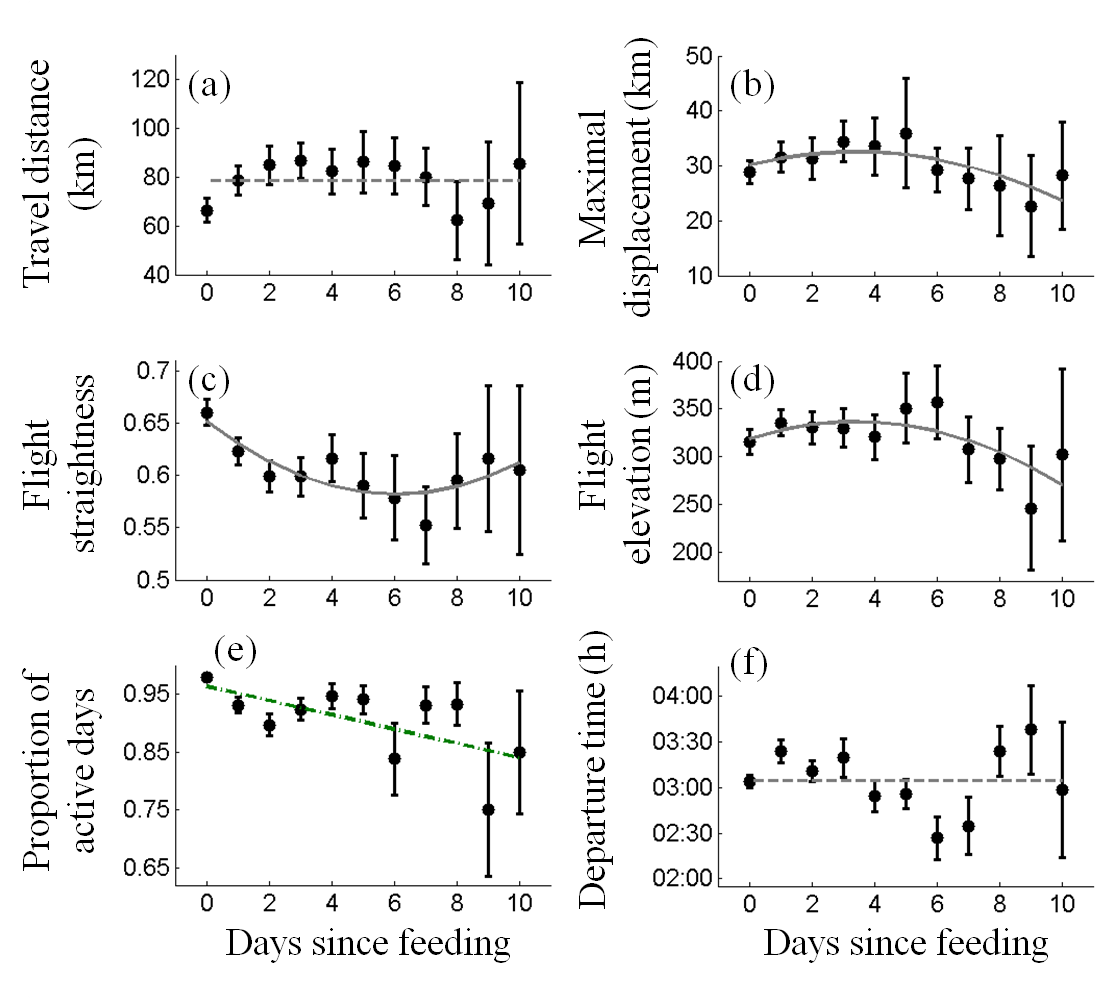

Figure S2.** The change in various movement characteristics of vultures as a function of time since the last feeding event (the days within a FDP) for events of vultures feeding on occasional carcasses outside the feeding stations. Each FDP sequence commences with a feeding event and ends one day before the successive feeding event. The lines represent the best fitting model among a hump-shaped model (second-order polynomial, solid line), a linear response model (first-order polynomial; green dash-dotted line), and a lack of response model (dashed line on the mean value). Models were ranked according to AIC_c_. FDP length had a hump-shaped effect on maximal displacement, flight elevation, a U-shaped effect on flight straightness, a linear effect on the proportion of active days and no effect on roost departure time and travel distance. Error bars are S.E. between individual mean values.
