# Supplementary material for: Mixed strategies of griffon vultures’ (Gyps fulvus) response to food deprivation lead to a hump-shaped movement pattern
Source: Mov Ecol. 2013 Jul 5;1(1):5. doi: 10.1186/2051-3933-1-5 (PMC4337378; doi:10.1186/2051-3933-1-5)
Supplement: Supplementary file 3 — Additional file 3: The effect of FDP length on vulture movement characteristics (Tables S2-S3). Three alternative models were considered: No effect, a linear effect, and a hump-shaped effect. The tables present the best fitting model for each variable using AICc and adjusted R2 estimation of goodness of fit. Table S2 presents the effect for the whole dataset and Table S3 for a subset of long FDPs (>=6). (DOCX 16 KB) [file 40462_2013_5_MOESM3_ESM.docx]

**Additional file 3: The effect of FDP length on vulture movement characteristics (Tables S2-S3)**

**Table S2**. The effect of FDP length on vulture movement characteristics. Three alternative models were considered: No effect, a linear effect, and a hump-shaped effect. The table presents the best fitting model for each variable using AIC_c_ and adjusted R^2^ estimation of goodness of fit.

| Variable (*x*) | Equation | AIC_c_ | Adj R^2^ |
| --- | --- | --- | --- |
| Daily travel distance (km) | $y=-0.8x^{2}+10.3x+51.4$ | 80.1 | 0.51 |
| Maximal displacement (km) | $y=-0.4x^{2}+3.9x+20.8$ | 57.6 | 0.63 |
| Flight elevation (m above ground) | $y=-1.1x^{2}+11.2x+302$ | 92.7 | 0.45 |
| Flight straightness | $y=0.613$ | 48.8 |  |
| Activity level (% of active days) | $y=91$ | 62.1 |  |
| Roost departure time (h after sunrise) | $y=3.133$ | 32.4 |  |

**Table S3**. The effect of FDP length on vulture movement characteristics during sequences of long FDP (>=6). Three alternative models were considered: No effect, a linear effect, and a hump-shaped effect. The table presents the best fitting model for each variable using AIC_c_ and the adjusted R^2^ goodness of fit.

| Variable (*x*) | Equation | AIC_c_ | Adj R^2^ |
| --- | --- | --- | --- |
| Daily travel distance (km) | $y=-0.8x^{2}+11.1x+36.1$ | 77.6 | 0.67 |
| Maximal displacement (km) | $y=-0.3x^{2}+4.3x+15.5$ | 62.3 | 0.47 |
| Flight elevation (m above ground) | $y=-1.1x^{2}+16.1x+258.1$ | 88.8 | 0.58 |
| Flight straightness | $y=-0.004x+0.619$ | 52.8 | 0.16 |
| Activity level (% of active days) | $y=90$ | 67.3 |  |
| Roost departure time (h after sunrise) | $y=3.13$ | 33.0 |  |
